# The Future of Toxicity Testing for Environmental Contaminants

**DOI:** 10.1289/ehp.12891

**Published:** 2009-07

**Authors:** Melissa G. Kramer, Michael Firestone, Robert Kavlock, Harold Zenick

**Affiliations:** Office of the Science Advisor, U.S. Environmental Protection Agency, Washington, DC, E-mail: Kramer.melissa@epa.gov; Office of Children’s Health Protection and Environmental Education, U.S. Environmental Protection Agency, Washington, DC; Office of Research and Development, U.S. Environmental Protection Agency, Research Triangle Park, NC

Toxicity testing and assessment sit on the cusp of a transformational change brought about by the rapid emergence of tools and capabilities in molecular biology and computational and informational sciences. This transformation has the potential to dramatically reshape the philosophy and approaches underlying toxicity testing and the assessment of human health risks associated with exposure to environmental contaminants.

Such a transformation is especially significant for agencies that are responsible for implementing congressionally mandated programs under which the risks of exposure to a wide variety of environmental pollutants are assessed and regulated. Most often, such regulatory decisions have relied on toxicity testing data obtained nearly exclusively from experimental animal models. This approach, however, presents challenges in accommodating the need for more efficient and cost-effective means to screen and prioritize chemicals for testing and addressing increasingly complex issues such as life-stage susceptibility and genetic variations in the human population, the risks of concurrent, cumulative exposure to multiple and diverse chemicals, and, fundamental to all, improved understanding of the mechanism through which toxicity occurs.

The U.S. Environmental Protection Agency (EPA) has recognized the potential application of emerging science to improve toxicity testing and risk assessment (U.S. EPA 2002U.S. EPA 2004), notably by taking the lead in commissioning the National Research Council (NRC) in 2004 to review existing strategies (NRC 2006) and develop a long-range vision for toxicity testing and risk assessment (NRC 2007). Beyond EPA, other federal programs have also recognized the need for this transformative shift, as reflected in the National Toxicology Program’s (NTP) *A National Toxicology Program for the 21st Century: Roadmap for the Future* (NTP 2004) and the Food and Drug Administration’s *FDA’s Critical Path Initiative* (FDA 2008).

To build on the NRC document, the U.S. EPA established an internal, cross-agency workgroup that produced *The U.S. Environmental Protection Agency’s Strategic Plan for Evaluating the Toxicity of Chemicals* (U.S. EPA 2009) to provide a framework for EPA to comprehensively move forward to incorporate this new scientific paradigm into future toxicity testing and risk assessment practices. The strategy is centered on three interrelated issues: *a*) the use of toxicity pathways information in screening and prioritization of chemicals for further testing; *b*) the use of toxicity pathways information in risk assessment; and *c*) organizational transition. The last element explicitly recognizes that regulatory offices within EPA will need to be actively involved in overseeing the significant transition to this new paradigm and the translation of the attendant data for regulatory application.

Research to address the first issue will build on the efforts of EPA’s ToxCast program in identifying and developing simple, reliable screening models to predict chemical hazard (U.S. EPA 2008a). The second effort will seek to apply the toxicity pathways concept in a systems biology approach, to better delineate the molecular and cellular changes that perturb normal homeostatic mechanisms toward a given toxicity pathway or set of toxicity pathways. This information should reduce the uncertainty currently associated with dose–response models by increasing their biological plausibility.

Recognizing the necessity and benefits of collaboration to achieve the NRC’s vision, EPA recently signed a Memorandum of Understanding with the NTP and the National Institutes of Health Chemical Genomics Center (U.S. EPA(2008b) to advance the high throughput screening and toxicity pathway profiling in risk assessment. This “Tox21” consortium is now actively coordinating efforts to identify chemicals, pathways, screening assays, and informatic approaches to assess the effects of thousands of chemicals (Kavlock et al. 2009). The U.S. EPA is also working with the European Commission and the Organization for Economic Cooperation and Development to facilitate global collaborations.

As recognized by the NRC (2007), the development and implementation of a transformational paradigm will require a major commitment to new funding to sustain an iterative and long-term process that changes institutional toxicity testing and risk assessment practices. Regulators, stakeholders, and the public must be confident that the new types of data can be used to effectively assess risk and ultimately protect public health. As such, education and transparent communication will be critical. Ultimately, the testing paradigm must be evaluated via a comprehensive development and review process, involving public comment, expert peer review, and harmonization with other agencies and international organizations. EPA’s *Strategic Plan for Evaluating the Toxicity of Chemicals* (U.S. EPA 2009) lays the framework upon which the development, implementation, acceptance, and application of this transformative paradigm can be built.

## Figures and Tables

**Figure:**
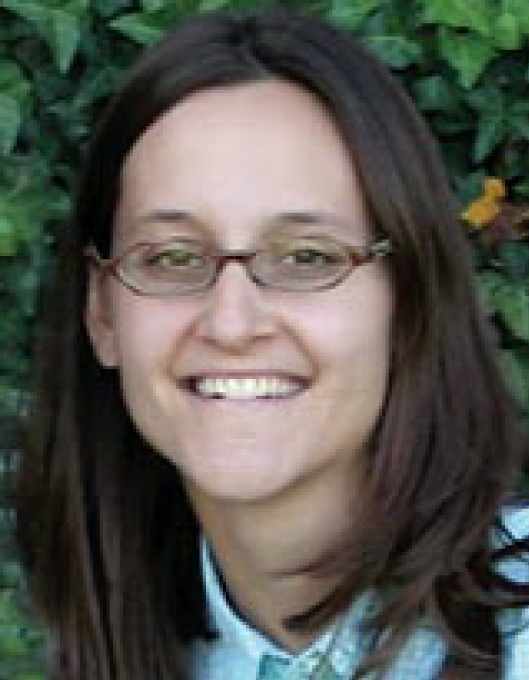
Melissa G. Kramer

**Figure:**
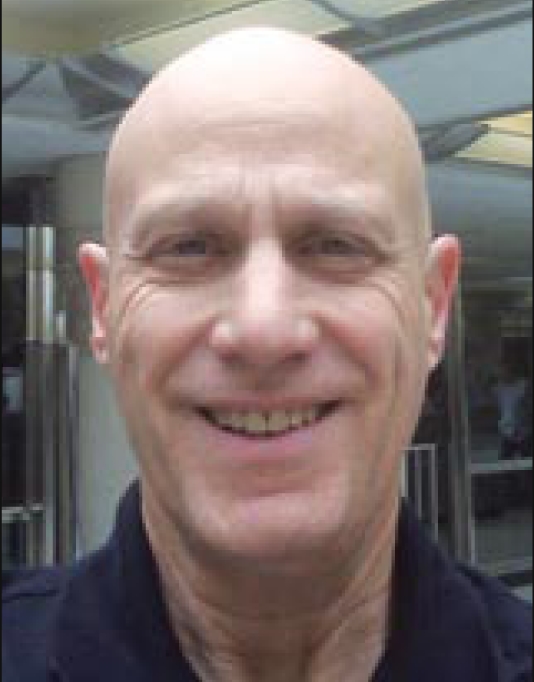
Michael Firestone

**Figure:**
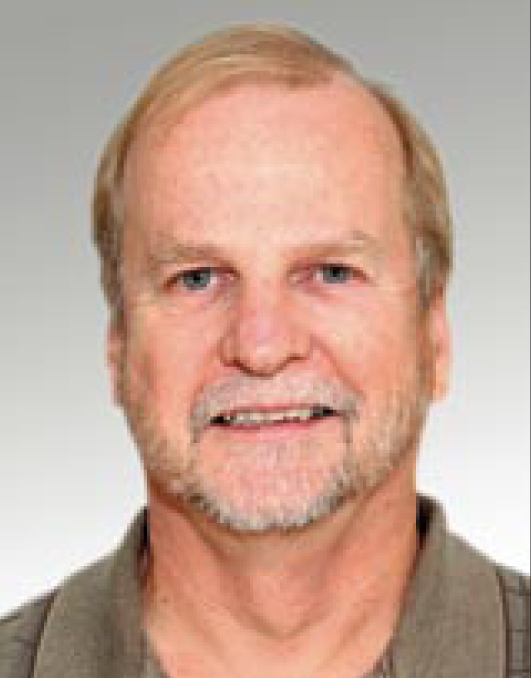
Robert Kavlock

**Figure:**
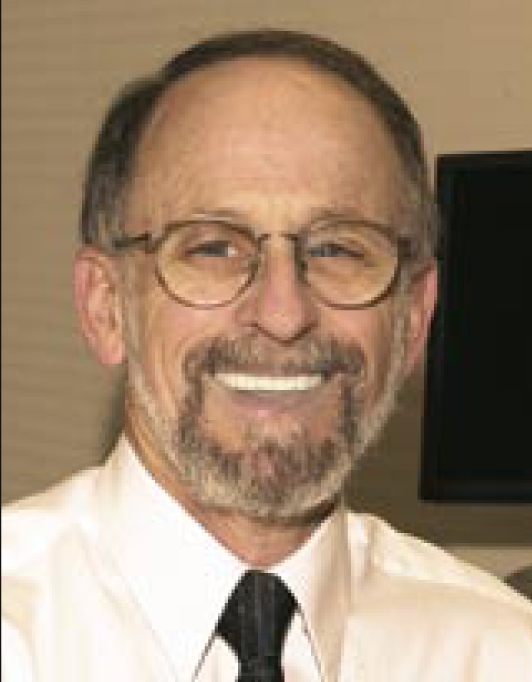
Harold Zenick

